# Hydroalcoholic Leaf Extract of *Isatis tinctoria* L. *via* Antioxidative and Anti-Inflammatory Effects Reduces Stress-Induced Behavioral and Cellular Disorders in Mice

**DOI:** 10.1155/2022/3567879

**Published:** 2022-06-25

**Authors:** Noemi Nicosia, Inga Kwiecień, Justyna Mazurek, Kamil Mika, Marek Bednarski, Natalizia Miceli, Salvatore Ragusa, Halina Ekiert, Michael Maes, Magdalena Kotańska

**Affiliations:** ^1^Department of Pharmacological Screening, Jagiellonian University, Medical College, Medyczna 9, PL 30-688 Kraków, Poland; ^2^Foundation “Prof. Antonio Imbesi”, University of Messina, Piazza Pugliatti 1, 98122 Messina, Italy; ^3^Department of Chemical, Biological, Pharmaceutical, And Environmental Sciences, University of Messina, Viale Ferdinando Stagno d'Alcontres 31, 98166 Messina, Italy; ^4^Chair and Department of Pharmaceutical Botany, Jagiellonian University, Medical College, Medyczna 9, PL 30-688 Kraków, Poland; ^5^Department of Health Sciences, University “Magna Graecia” of Catanzaro, V. Europa, IT-88100 Catanzaro, Italy; ^6^Department of Psychiatry, Faculty of Medicine, Chulalongkorn University, Bangkok, Thailand; ^7^Department of Psychiatry, Medical University of Plovdiv, Plovdiv, Bulgaria; ^8^IMPACT Strategic Research Center, Deakin University, Geelong, Australia

## Abstract

Stress that can occur at different levels of a person's life can cause and exacerbate various diseases. Oxidative stress and inflammation underlie this process at the cellular level. There is an urgent need to identify new and more effective therapeutic targets for the treatment of stress-induced behavioral disorders and specific drugs that affect these targets. *Isatis tinctoria* L. is a herbaceous species in the Brassicaceae family. Due to its potential antioxidant, nitric oxide- (NO-) inhibiting, anti-inflammatory, and neuroprotective properties, *I. tinctoria* could be used to treat depression, anxiety, and stress resistance. Hence, the present study is aimed at delineating whether administration of *I. tinctoria* leaf extract may improve stress-induced disorders in mice. A set of four behavioral tests was selected that together are suitable for phenotyping acute restraint stress-associated behaviors in mice, namely locomotor activity, social integration, dark/light box, and splash tests. The plasma and brains were collected. A brain-derived neurotrophic factor, tumor necrosis factor-alpha, C-reactive protein, corticosterone, NO, reactive oxygen species levels, superoxide dismutase and catalase activity, and ferric-reducing antioxidant power were measured. In mice stressed by immobilization, decreased locomotor activity, anxiety-like behavior, and contact with other individuals were observed, as well as increased oxidative stress and increased levels of nitric oxide in the brain and plasma C-reactive protein. A single administration of *I. tinctoria* leaf extract was able to reverse the behavioral response to restraint by a mechanism partially dependent on the modulation of oxidative stress, neuroinflammation, and NO reduction. In conclusion, *Isatis tinctoria* hydroalcoholic leaf extract can reduce stress-induced behavioral disturbances by regulating neurooxidative, neuronitrosative, and neuroimmune pathways. Therefore, it could be recommended for further research on clinical efficacy in depression and anxiety disorder treatment.

## 1. Introduction

Depression, anxiety, and stress-related disorders are the most prevalent psychiatric disorders, accounting for a disproportionate share of mental illnesses in highly developed countries [1]. Stress levels throughout a person's life may contribute to the development of a variety of diseases, both central and peripheral (hypertension, ischemic heart disease, muscle spasms with subsequent chronic pain, cancer, inflammation, and weight disorders), and are often fatal in many people [2–6]. Anxiety and depression have raised to prominence as major health problems on a global scale in recent years. As a result, there is an urgent need to identify new and more effective therapeutic goals for the treatment of stress-induced behavioral disorders, as well as specific drugs that affect these goals. It is critical not only from a medical but also from a socioeconomic standpoint.

Newer findings suggest the crucial role that the immune system, oxidative, and nitrative stress play in the development of psychiatric disorders such as anxiety and major depression [7].

It is well known that brain tissue is particularly susceptible to oxidative damage, compared to other organs, due to the relatively high content of iron and peroxide fatty acids, in addition to its limited antioxidative capacity [8]. Reactive oxygen species are produced during physiological processes, but when present in excess or under conditions of decreased availability of antioxidant defenses, they can cause structural and functional changes that produce cell injury [9].

NO is a messenger molecule that is widely distributed in cells and can influence a wide variety of physiological and pathological processes. Overproduction of NO causes tissue damage and can cause various inflammatory diseases. However, NO also plays an important neuromodulatory role in the central nervous system. It is synthesized from L-arginine by nitric oxide synthase and is involved in neurotransmission, synaptic plasticity, learning, pain sensation, aggression, stress response, and depression [10]. Some evidence has shown that lowering nitric oxide levels in the hippocampus can induce antidepressant-like effects by increasing stress-coping activity, thereby confirming that endogenous hippocampal nitric oxide plays a role in the neurobiology of stress and depression [7, 11]. Literature data indicate that nitric oxide synthase inhibitors increased extracellular serotonin and dopamine levels in the rat ventral hippocampus, while L-arginine acted the opposite, thereby demonstrating the role of endogenous nitric oxide in regulating serotonin and dopamine levels in the hippocampus [12].

One of the most recent and important findings is that the elements of the immune system that mediate inflammation may be closely related to behavioral disorders induced by stress. Inflammation is usually viewed as the body's primary response to physical damage or infection. However, there is now substantial evidence that mental stress can cause a significant increase in inflammation. In turn, worsening inflammation can trigger profound behavioral changes that include the initiation of depressive symptoms such as sad mood, anhedonia, fatigue, psychomotor retardation, and sociobehavioral withdrawal [13].

Different nutraceuticals, including flavonoids, show antidepressant activities and may reduce stress-induced behaviors in animal models and humans as well [7, 14, 15].

*Isatis tinctoria* L. (woad) is an herbaceous species in the Brassicaceae family that is widely distributed throughout the world and has been valued for centuries for its dyeing properties. The root and leaves have a long and well-documented history of use as a medicinal raw material in both Western and Eastern cultures [16, 17]. The *I. tinctoria* root also has monographs in both the European and Chinese pharmacopeias. Today, it is widely used in Traditional Chinese Medicine (TCM) to treat a variety of diseases, including hepatitis, influenza, and bacterial infections of the respiratory system [18].

The interest in this plant has rapidly grown due to its phytochemical profile, rich in bioactive compounds including glucosinolates, flavonoids, and indole, quinazolone, and quinoline alkaloids, which have anti-inflammatory, antitumor, antimicrobial, analgesic, and antioxidant properties [16]. Numerous phytochemical studies of the root and leaves of *I. tinctoria* indicate differences in their chemical composition. Depending on the extraction and separation methods, particular groups of metabolites are dominant [19, 20]. In in vitro and in vivo studies both the root and leaf extracts from *I. tinctoria* show anti-inflammatory properties by inhibiting proinflammatory cytokines [21, 22] and suppression of nitric oxide (NO) production, which at least in part explain its impact on inflammatory and oxidative pathways [23–25]. *Isatis tinctoria* antioxidants' effects against free radicals are further supported by many in vitro assays revealing its strong scavenging activity as well as its good reducing power [16]. More recent research has focused on its neuroprotective properties, for example, against hydrogen peroxide-induced cell injury (H_2_O_2_) [26].

Due to the high concentration of flavonoids and other phenolic compounds in *I. tinctoria* leaves, which have antioxidant, NO inhibitory, anti-inflammatory, and neuroprotective properties, the extract of this plant may be useful to reduce depressive and anxiety symptoms, as well as improve resilience to stress, potentially providing an alternative to conventional antidepressants. Nevertheless, to date, no studies have examined the effects of treatment with *I. tinctoria* on depressive-like behaviors. Therefore, this study was carried out to delineate the effects of *I. tinctoria* on stress-induced behavioral disorders in mice.

## 2. Materials and Methods

### 2.1. Plant material

Cauline leaves were picked from *Isatis tinctoria* L. growing wild around Acireale (Catania, Sicily, Italy). The taxonomic identification of the plant materials was confirmed by Prof. Salvatore Ragusa; specimens are deposited in the Herbarium of the Department of Scienze della Salute, University “Magna Graecia” of Catanzaro (Italy), under accession number no. 327/11. The dried plant material was extracted in accordance with the procedure reported by Taviano et al. [27]. Finally, the hydroalcoholic extract (MeOH 70%) was evaporated to dryness *in vacuo*, and the yield, referred to as 100 g of dried plant material, was 24.32%. The phytochemical analysis of the extract was previously performed by HPLC-PDA-ESI-MS [27].

### 2.2. Extract and Chemicals

Three different concentrations of *I. tinctoria* leaf extract (IT) were tested *in vivo*. To prepare the solution for injecting mice, dried extracts of *I. tinctoria* leaves were suspended in 1% Tween 80 solution (Sigma-Aldrich, France) to obtain the final concentration. The solutions were freshly prepared before the experiment and administered in a single dose to mice intraperitoneally (i.p.) at the following doses: 50 mg/kg, 100 mg/kg, or 500 mg/kg b.w. Vehicle (1% Tween 80) was administered i.p. at a volume of 10 mL/kg. Bupropion at a dose of 10 mg/kg b.w. was chosen as the reference compound [47]. Bupropion inhibits dopamine reuptake and increases its availability in the synaptic cleft. Dopamine is associated with motivation, reward, and hedonic states, and therefore, increasing dopamine levels could improve depressive-like symptoms [28]. In our study, we chose bupropion because it is suggested to exert antidepressant effects and it is expected to reduce oxidative stress [29] and inflammation [30]. This drug was used in the treatment of posttraumatic stress disorder [31].

Heparin was purchased from Polfa Warszawa S.A. (Warsaw, Poland).

### 2.3. Animals

Six-week-old, male Albino Swiss mice, CD-1, weighing 20–22 g, were used. The animals (160 subjects) were obtained from the Animal House of the Pharmaceutical Faculty of Jagiellonian University and kept in environmentally controlled rooms, in standard cages lit by artificial light for 12 hours each day. Animals were free to access food and water, except for the time of the acute experiment. The randomly established experimental groups consisted of 8 mice. All animal care and experimental procedures were carried out in accordance with European Union and Polish legislation acts concerning animal experimentation and were approved by the Local Ethics Committee at the Jagiellonian University in Cracow, Poland (No.: 473/2020, 511/2021, 545B/2021).

### 2.4. Behavioral Studies

Behavioral studies were carried out in a quiet and stress-free environment, and the animals were handled several days before the actual study to acquaint them with the experimenter. The temperature and lighting conditions of the facility were kept constant. Mice are more active during the dark phase of their light-dark cycle, and so, the animals were tested at approximately the same time of day for better within-study and cross-study comparisons.

**Figure 1:**
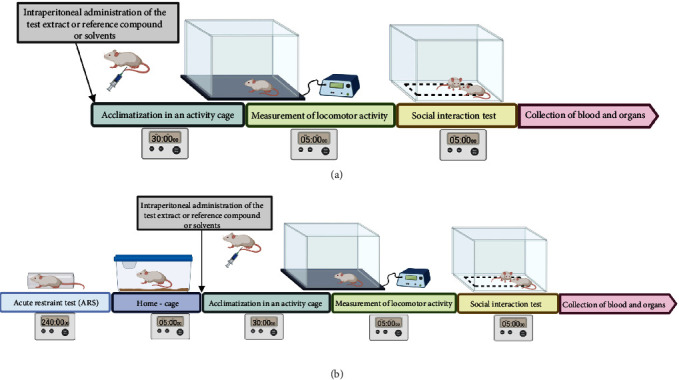
Scheme of locomotor activity test and social integration test (a) without the ARS model and (b) after the ARS model.

**Figure 2:**
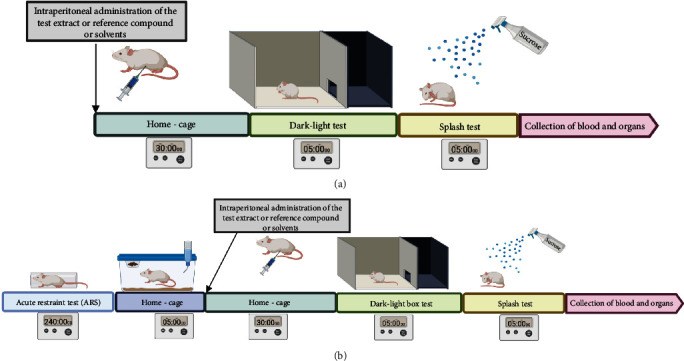
Scheme of dark/light box test and splash test (a) without the ARS model and (b) after the ARS model.

All scheduled tests are mild on animals (light, pain-free). Therefore, it was planned to carry out the tests in pairs in one mouse to save the number of animals in accordance with the recommendations of the Ethics Committee for Animal Research. Thus, spontaneous activity was assessed 5 minutes before the social integration test, whereas the dark/light box test was run 5 minutes before the splash test. The experiments schemes are shown in Figures 1 and 2.

#### 2.4.1. Locomotor Activity Test

Locomotor activity was individually recorded for each animal using specifically designed activity cages made of clear Perspex (40 cm × 40 cm × 31 cm, Activity Cage 7441, Ugo Basile, Italy) [32]. The cages came supplied with I.R. horizontal beam emitters connected to a counter which records the light-beam interruptions. The prepared suspensions were administered as i.p. injections 30 min before testing. The control animals received i.p. injections of vehicles. Each mouse was placed in a cage for a 30-minute habituation period. After that time, the number of breaks in the photobeams was measured for 5 minutes which is the same time as the observation period used in other tests.

#### 2.4.2. Social Integration Test

The social integration test was carried out according to a procedure described by Kraeuter and colleagues with minor modifications [33].

The test mouse was placed into the center zone of the open-field box (light-grey colored 42 × 42 × 42 cm polyvinyl chloride (PVC) chamber with a marked 20.5 × 20.5 central zone), immediately before placing the stimulus mouse in the same area of the box. Mice behaviors were recorded by the camera for 5 minutes. The number of approaches of the test mouse versus stimulus mouse was counted.

#### 2.4.3. Dark/Light Box Test

The dark/light box test was carried out according to a procedure described by Bourin and Hascoet and Kulesskaya and Voikar with minor modifications [34, 35]. The test was carried out in the open-field arena with a white floor (40 cm × 40 cm × 31 cm, Activity Cage 7441, Ugo Basile, Italy) equipped with infrared light sensors (at 1.5 cm intervals) detecting horizontal and vertical activity. The dark insert (with black walls and lid, nontransparent for visible light) was used to divide the arena into two parts of size: 40 × 13 cm (dark box) and 40 × 27 cm (light box). An opening (width 3.5 cm and height 5 cm) in the wall of the insert allows animals' free movement from one compartment to another. The light side was illuminated (illumination in the center of the light compartment ~1000 lx). The animal was released in the center of the light compartment (facing away from the opening) and allowed to explore the arena for 5 min. The distance traveled in light, the number of entrances to the dark box, and the time spent in different compartments were recorded.

#### 2.4.4. Splash Test

The splash test was carried out in a standard mouse cage according to a procedure described by Casaril and colleagues [36].

Briefly, animals were splashed on the dorsal coat with 10% sucrose solution to induce grooming behavior, defined as cleaning of the fur by licking, scratching, or head washing. The latency of the first grooming and the total grooming time were measured for 5 min immediately after the splash of sucrose solution and scored by trained personnel blinded to the treatments.

#### 2.4.5. Acute Restraint Stress (ARS) Model

The physical restraint was performed in mice as previously reported by Casaril and colleagues [36].

Briefly, mice were subjected to immobilization for 240 min using an individual rodent restraint device made of Plexiglas, restraining all physical movement and causing no pain. Mice were deprived of food and water during physical stress. After restraint stress, the mice were put back in their home cage, and 5 minutes later, they received tested extracts, vehicle, or reference compound. They were subjected to behavioral tests 30 minutes later.

### 2.5. Collecting Plasma and Brains

At the end of the experiment, 60 min after administration IT, vehicle, or bupropion and 20 min after i.p. administration of heparin (2500 units/mice), the blood was collected after decapitation and then centrifuged at 600 × g (15 min, 4°C) to obtain plasma. The brain (only the cerebral cortex with the hippocampus) was collected on ice immediately after decapitation and freezing in liquid nitrogen and then placed in a freezer (-80°C) until biochemical assays are performed. On the day of the assays, brain parts were weighed, and homogenates were prepared by homogenization at the ratio of 1 g of the tissue in 4 ml of 0.1 M phosphate buffer, pH 7.4 using an IKA-ULTRA-TURRAX T8 homogenizer.

### 2.6. Biochemical Analysis

For the determination of brain-derived neurotrophic factor (BDNF), tumor necrosis factor-alpha (TNF-*α*), or C-reactive protein (CRP) levels, standard ELISA tests with spectrophotometric reading (Shanghai Sunred Biological Technology Co., Ltd, China) were applied. For the determination of superoxide dismutase (SOD) and catalase (CAT) activities and corticosterone or nitric oxide (NO) levels, standard enzymatic and spectrophotometric tests were used (CaymanChem, USA).

#### 2.6.1. Reactive Oxygen Species (ROS)

ROS were assayed according to the method of Bondy and Guo [37], using DCFH-DA which is deesterified in brain homogenates to 2 ′,7 ′-dichlorofluorescein acid and then oxidized by ROS to fluorescent 2 ′,7 ′-dichlorofluorescein (DCF). Briefly, to 10 *μ*l of homogenate, 990 *μ*l of 0.1 M phosphate buffer (pH 7.4) and 10 *μ*l of 1.25 M DCFH-DA dissolved in ethanol were added. The reaction mixture was incubated at 37°C for 30 min, protecting the samples from light. The measurements were conducted using a fluorometer at wavelengths: *A*_ex_ = 488 nm and *A*_em_ = 525 nm. ROS were evaluated using a standard curve for 1 *μ*M DCF.

#### 2.6.2. Ferric-Reducing Antioxidant Power (FRAP) assay

The assay was performed according to Benzie and Strain [38] with some modifications. The FRAP working solution was prepared before the start of the analysis: 0.3 mol acetate buffer (pH 3.6), 0.01 mol TPTZ (2,4,6-tripyridyl-s-triazine; Sigma-Aldrich) in 0.04 mol HCl (POCh, Poland), and 0.02 M FeCl_3_ × 6H_2_O in water (iron (III) chloride hexahydrate; Chempur, Poland) were mixed in a volumetric ratio of 10 : 1 : 1 and protected from light.

Next, 20 *μ*l of the plasma sample tested, brain homogenate, or FeSO_4_ × 7H_2_O solution was mixed with 180 *μ*l of the FRAP working solution. The mixtures obtained were incubated at 37°C for 30 minutes, and their absorbance was measured at 593 nm. FeSO_4_ × 7H_2_O (100–1000 *μ*M/l) was used for a calibration curve. Deionized water with FRAP solution was used as a blank.

### 2.7. Statistical Analysis

Statistical calculations were performed using GraphPad Prism 6 software (GraphPad Software, USA). The results are expressed as mean + Δ/2, where Δ is a width of the 95% confidence interval (CI), *n* = 6 − 8. Statistical significance was calculated using one-way ANOVA, Dunnett's, or Tukey post hoc test (respectively, when there are one or two control groups). Differences were considered statistically significant at  ^∗^*p* ≤ 0.05,  ^∗∗^*p* ≤ 0.01, and  ^∗∗∗^*p* ≤ 0.001.

## 3. Results

### 3.1. Influence of IT on Locomotor Activity

**Figure 3:**
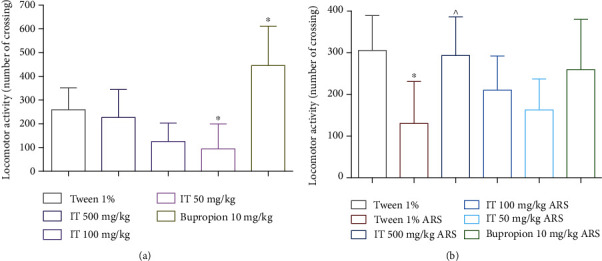
The effect of *Isatis tinctoria* leaf extract on locomotor activity in mice: (a) without stress (naïve mice), (b) after stress. ARS: acute stress model, IT: *Isatis tinctoria* leaf extract. The results are presented as the mean of the measurements ± Δ/2, where Δ is the width of the 95% confidence interval (CI). One-way ANOVA was used to calculate the significance of differences between groups, followed by Dunnett's or Tukey's post hoc test, *n* = 7 − 8.  ^∗^Significant difference vs. the control group without stress. ∧Significant difference vs. the control group with stress. significance level:  ^∗,^∧*p* < 0.05.

IT administered at a dose of 50 mg/kg body weight (b.w.) significantly (*p* < 0.05) decreased the locomotor activity, compared to the activity determined in the control group. The remaining doses 500 mg/kg b.w. and 100 mg/kg b.w. did not cause a significant effect on the locomotor activity. The reference compound bupropion, administered at a dose of 10 mg/kg b.w., significantly (*p* < 0.05) increased the locomotor activity. The results are shown in Figure 3(a).

In the acute restraint stress (ARS) model, in stressed control mice, locomotor activity was significantly (*p* < 0.05) lower than in the naïve control group. However, this significant decrease in activity was not observed in all groups administered IT or the reference compound. In addition, in the group administered with the highest dose (500 mg/kg b.w.), locomotor activity was significantly augmented (*p* < 0.05) compared to the stressed control group. The results are shown in Figure 3(b).

### 3.2. Influence of IT on Incidents of Social Interaction

**Figure 4:**
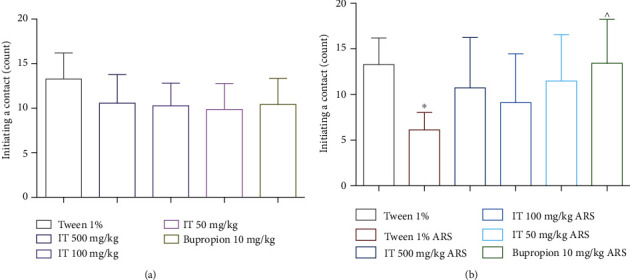
The effect of *Isatis tinctoria* leaf extract on the number of contacts initiated by test mice: (a) without stress (naïve mice), (b) after stress. ARS: acute stress model, IT: *Isatis tinctoria* leaf extract. The results are presented as the mean of the measurements ± Δ/2, where Δ is the width of the 95% confidence interval (CI). One-way ANOVA followed by Dunnett's or Tukey's post hoc test was used to calculate the significance of differences between the groups, *n* = 7 − 8.  ^∗^Significant difference vs. the control group without stress. ∧Significant difference vs. the control group with stress. Significance level:  ^∗,^∧*p* < 0.05.

In naïve mice, all doses of IT tested (500 mg/kg b.w., 100 mg/kg b.w., and 50 mg/kg b.w.) as well as bupropion did not significantly affect the number of approaches of the test mice versus stimulus mice. The results are shown in Figure 4(a).

In the ARS model, the number of approaches initiated by the mice in the stressed control group was significantly lower (*p* < 0.05) compared to the naïve control group. For all tested doses of IT and bupropion, an increase in the number of approaches initiated by the test mice towards the stimulus mice was observed compared to the stressed control group (no significant differences compared to the nonstress control group). However, only bupropion increased the number of contacts initiated at a significant level (*p* < 0.05). The results are shown in Figure 4(b).

### 3.3. Influence of IT on Behaviors in an Unknown Space (Dark/Light Box Test)

**Figure 5:**
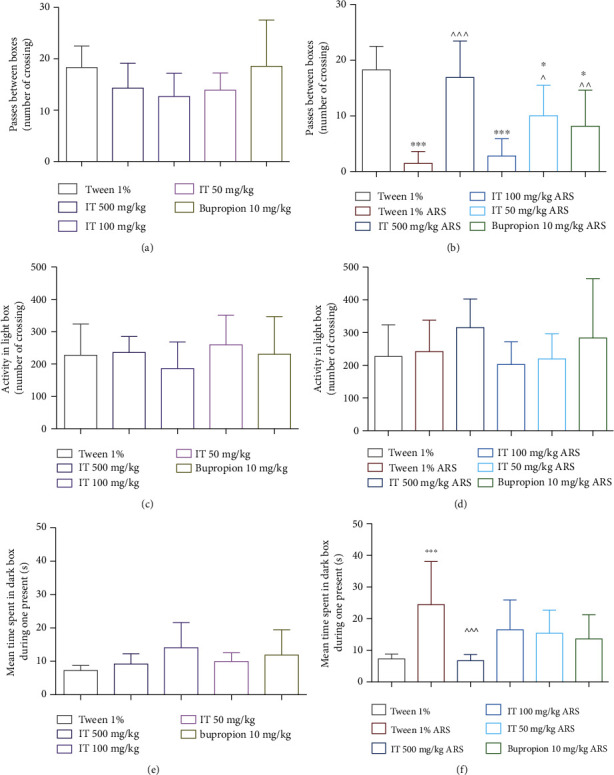
The effect of *Isatis tinctoria* leaf extract on behavior in an unknown space (dark/light box test). The number of passes through the hole between light and dark fields: (a) without stress (naïve mice), (b) after stress. Locomotor activity: (c) without stress (naïve mice), (d) after stress. The time spent by mice in the dark field of the activity cage during one stay: (e) without stress, (f) after stress. ARS: acute stress model, IT: *Isatis tinctoria*.

In naïve mice, all the IT-treated and bupropion-treated groups showed no significant changes in the number of passes through the hole between light and dark fields. The results are shown in Figure 5(a).

The results are presented as the mean of the measurements ± Δ/2, where Δ is the width of the 95% confidence interval (CI). One-way ANOVA followed by Dunnett's or Tukey's post hoc test was used to calculate the significance of differences between the groups, *n* = 7 − 8.  ^∗^Significant difference vs. the control group without stress. ^∧^Significant difference vs. the control group with stress. Significance level:  ^∗,^∧*p* < 0.05; ∧∧*p* < 0.01;  ^∗∗∗,^∧∧∧*p* < 0.001.

In the ARS model, the number of transitions between light and dark box was significantly lower (*p* < 0.001) in the stressed control mice than in the naïve control group. IT at a dose of 100 mg/kg b.w. had no significant effect on the number of passes; the number of counts in this group was comparable to the number of counts recorded in the stressed control group. After administration of a dose of 50 mg/kg b.w. or bupropion, there were significantly fewer passes compared to the number of passes observed in the naïve control group. In addition, after administration of IT in doses of 500 mg/kg b.w. and 50 mg/kg b.w. or bupropion, the nember of light-field and dark-field passes significantly increased compared to the number of passes in stressed control mice (*p* < 0.001, *p* < 0.05, and *p* < 0.01, respectively). The results are shown in Figure 5(b).

IT administered to mice at the following doses, 500 mg/kg b.w., 100 mg/kg b.w., 50 mg/kg b.w., or bupropion, did not significantly affect the locomotor activity of mice in light box, both in naïve and stressed control mice. The results are shown in Figures 5(c) and 5(d).

In naïve mice, in all IT-treated groups, no significant influence of the tested extracts was observed regarding the time spent by the mice in the dark field of the activity cage. The reference compound, bupropion, also did not significantly affect the time spent by mice in the dark. The results are shown in Figure 5(e).

In the ARS model, in the stressed control mice, the time spent in the dark field of the cage during one stay was statistically significantly longer (*p* < 0.001) than in the naïve control group. In all groups treated with IT or bupropion, the time spent in the dark field of the cage during one stay was shorter compared to the stressed control group (no significant differences compared to the control group without stress control group), whereas only at a dose of 500 mg/kg the reduction in time was significant, *p* < 0.001. The results are shown in Figure 5(f).

### 3.4. Influence of IT on Behaviors during Splash Test

**Figure 6:**
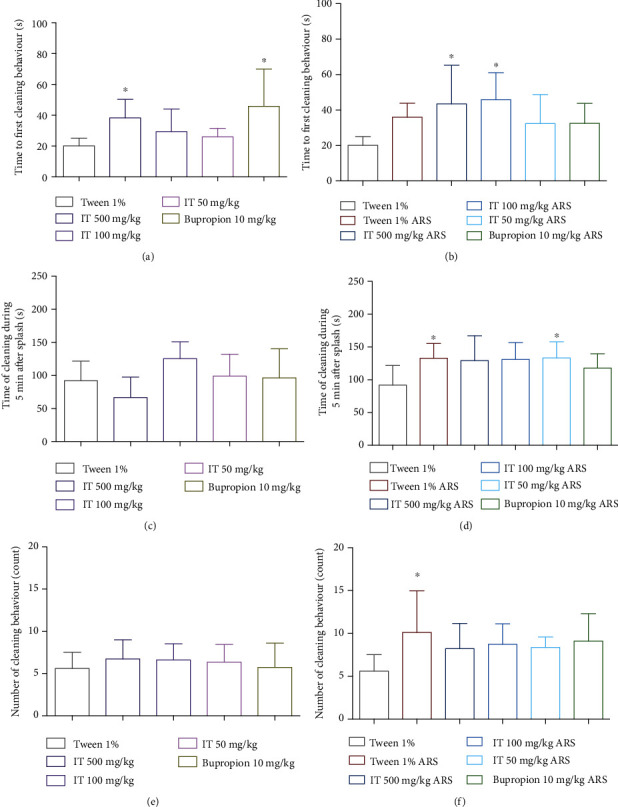
The effect of *Isatis tinctoria* leaf extract on behavior during splash test. The time to start washing by mice: (a) without stress (naïve mice), (b) after stress. The washing time of mice within 5 minutes: (c) without stress (naïve mice), (d) after stress. The number of washes: (e) without stress (naïve mice), (f) after stress. ARS: acute stress model, IT: *Isatis tinctoria*. The results are presented as the mean of the measurements ± Δ/2, where Δ is the width of the 95% confidence interval (CI). One-way ANOVA followed by Dunnett's or Tukey's post hoc test was used to calculate the significance of differences between the groups; *n* = 7 − 8.  ^∗^Significant difference vs. the control group without stress; significance level:  ^∗^*p* < 0.05.

In naïve mice, IT administered at the highest dose (500 mg/kg b.w.) significantly (*p* < 0.05) delayed the start of grooming behavior compared to the time measured for mice in the control group. The reference compound, bupropion, also significantly (*p* < 0.05) prolonged the time to start grooming in mice. The remaining IT doses, 100 mg/kg b.w. and 50 mg/kg b.w., did not significantly extend this time. The results are shown in Figure 6(a).

In stressed mice, no elongation of the interval between the splash of sucrose and the first grooming behavior was detected compared to the naïve control mice. All doses of IT or bupropion did not affect the time to start grooming compared to the stressed control group. IT at doses 500 mg/kg b.w. and 100 mg/kg b.w. significantly (*p* < 0.05) prolonged the time to the first grooming behavior compared to naïve control mice. IT dose 50 mg/kg b.w. or bupropion did not significantly extend this time. The results are shown in Figure 6(b).

In naïve mice, no significant effect of the tested extract was observed on the grooming time of the mice during the 5 min splashing test. The reference compound, bupropion, also did not significantly affect grooming time. The results are shown in Figure 6(c).

In the ARS model, in the stressed control mice, grooming time was significantly longer (*p* < 0.05) than in the naïve control group. In the groups with stress given IT at a dose of 50 mg/kg b.w., the grooming time of the mice compared to the naïve control group was also significantly longer (*p* < 0.05) and did not differ significantly from the level determined in the stressed control group. No significance was observed in the remaining groups given IT. Bupropion also did not exhibit a significant effect on this parameter. The results are shown in Figure 6(d).

In naïve mice, no significant influence of the tested extract on the number of washes was observed. The reference compound bupropion also did not significantly affect this number. The results are shown in Figure 6(e).

In the ARS model, the number of washes in the stressed control mice was significantly greater than in the naïve control group. After administration of IT at all doses tested (500 mg/kg b.w., 100 mg/kg b.w., and 50 mg/kg b.w.) as well as bupropion (10 mg/kg b.w.) to stressed mice, no significant change in the number of washes was observed both compared to the stressed control group but also compared to the naïve control group. The results are shown in Figure 6(f).

### 3.5. Influence of IT on Plasma Corticosterone Level

**Figure 7:**
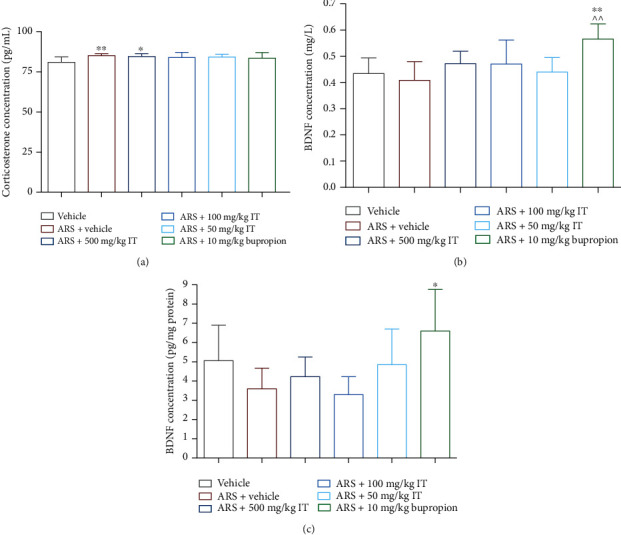
Levels of corticosterone or BDNF. (a) Plasma corticosterone, (b) plasma BDNF, and (c) brain BDNF (c). ARS: acute stress model, IT: *Isatis tinctoria*.

The plasma level of corticosterone in the stressed control mice was significantly greater than in the naïve control group. In the group treated with the highest dose (500 mg/kg b.w.) of IT, the plasma corticosterone level significantly (*p* < 0.05) elevated in comparison to the level determined in the naïve control group. There were no significant differences in the corticosterone levels in the plasmas of the stressed mice treated with IT at doses 100 or 50 mg/kg b.w. compared to the levels of both control groups. The results are shown in Figure 7(a).

The results are presented as the mean of the measurements ± Δ/2, where Δ is the width of the 95% confidence interval (CI). One-way ANOVA followed by Tukey's post hoc test was used to calculate the significance of differences between the groups; *n* = 7 − 8.  ^∗^Significant difference vs. control group without stress. ∧Significant difference vs. the control group with stress. Significance level:  ^∗^*p* < 0.05;  ^∗∗,^∧∧p < 0.01.

### 3.6. Influence of IT on Plasma or Brain BDNF Level

There were no significant differences in BDNF levels in the plasmas of the control groups. Significantly higher levels of plasma BDNF concentration were observed compared to plasma levels of both control groups only in the group treated with bupropion (*p* < 0.01). The results are shown in Figure 7(b). Significantly higher levels of BDNF were determined in the brain than in the brain of stressed mice only in the brain of mice treated with bupropion. The results are shown in Figure 7(c).

### 3.7. Influence of IT on Plasma CRP Level

**Figure 8:**
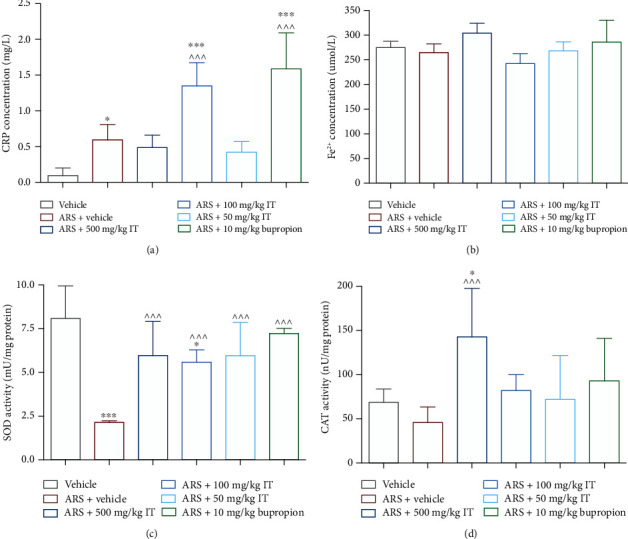
Plasma level of (a) CRP, (b) plasma total antioxidant activity, (c) plasma SOD activity, and (d) plasma CAT activity. ARS: acute stress model, IT: *Isatis tinctoria*. The results are presented as the mean of the measurements ± Δ/2, where Δ is the width of the 95% confidence interval (CI). One-way ANOVA followed by Tukey's post hoc test was used to calculate the significance of differences between the groups; *n* = 7 − 8.  ^∗^Significant difference vs. the control group without stress. ∧Significant difference vs. the control group with stress. Significance level:  ^∗^*p* < 0.05;  ^∗∗,^∧∧∧*p* < 0.001.

In the plasma collected from mice in the stressed control group, a significantly (*p* < 0.05) higher level of CRP was determined compared to the level determined in the plasma collected from the mice naïve control group. After administration of 100 mg/kg of IT, and also of bupropion, the plasma level of CRP also increased significantly compared to the level measured in the control groups (*p* < 0.001). There were no significant differences in plasma CRP levels in the groups treated with IT at doses of 500 or 50 mg/kg b.w. The results are shown in Figure 8(a).

### 3.8. Influence of IT on Antioxidant Parameters

**Figure 9:**
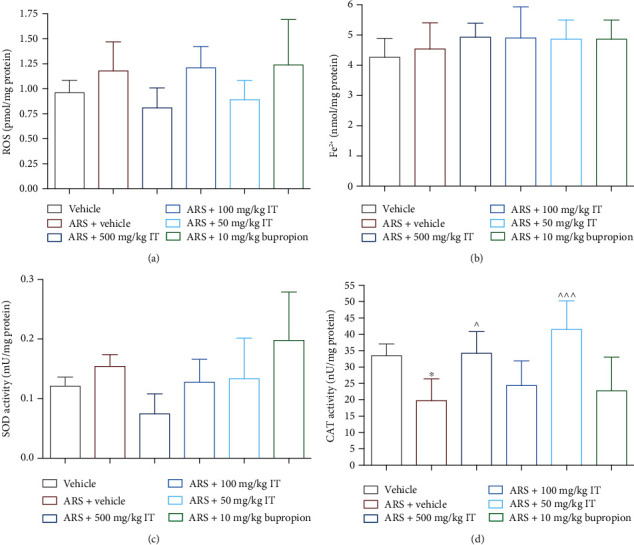
(a) Brian level of ROS, (b) brain total antioxidant activity, (c) brain SOD activity, and (d) brain CAT activity. ARS: acute stress model, IT: *Isatis tinctoria*. The results are presented as the mean of the measurements ± Δ/2, where Δ is the width of the 95% confidence interval (CI). One-way ANOVA followed by Tukey's post hoc test was used to calculate the significance of differences between the groups; *n* = 7 − 8.  ^∗^Significant difference vs. the control group without stress. ∧Significant difference vs. the control group with stress. Significance level:  ^∗,^∧*p* < 0.05;  ^∗∗,^∧∧∧*p* < 0.001.

No significant differences in ferric-reducing ability were determined in the plasmas and brains collected from mice in this experiment. The results are shown in Figures 8(b) and 9(b).

The activity of SOD or CAT in the plasma collected from the stressed control animals resulted lower than the activity of these enzymes measured in the plasma collected from the naïve control group. The difference in SOD activity was significant (*p* < 0.001). After administration of the extract or bupropion, the activities of these enzymes determined in plasma did not differ significantly from the levels determined in the naïve control group (except CAT activity in the group treated with 500 mg/kg b.w.). Plasma activities of SOD from the extract- or bupropion-treated groups were significantly higher than the SOD activity in the plasma taken from the previously stressed control group (*p* < 0.05). CAT activity in the plasma collected from the IT-treated group at a dose of 500 mg/kg was significantly higher than the activity determined in the stressed control group (*p* < 0.001) and in the naïve control group (*p* < 0.05). The results are shown in Figures 8(c) and 8(d).

No significant differences in ROS level were determined in brains collected from mice in this experiment. The results are shown in Figure 9(a).

There were no differences in SOD activity in the brains collected from the different groups of animals. The results are shown in Figure 9(c).

The activity of CAT in the brain from the stressed control animals was significantly lower (*p* < 0.05) than the activity of this enzyme measured in the brain collected from the naïve control group. CAT activities after IT treatment at doses of 50 mg/kg b.w. or 500 mg/kg b.w. were significantly higher than SOD activity in the brain taken from the stressed control group (*p* < 0.05 and *p* < 0.001). After administration of the extract at a dose of 100 mg/kg b.w. or bupropion, the CAT activities determined in plasma did not differ significantly from the levels determined in the naïve control group. The results are shown in Figure 9(d).

**Figure 10:**
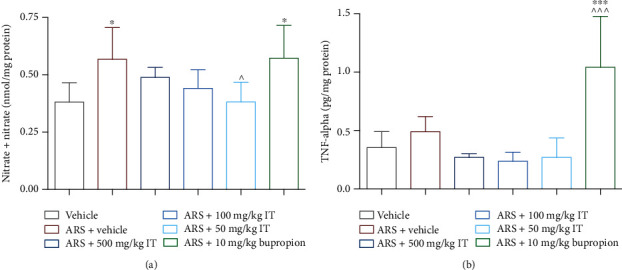
Levels of NO or TNF-alpha. (a) Brain NO, (b) brain TNF-alpha. ARS: acute stress model, IT: *Isatis tinctoria*. The results are presented as the mean of the measurements ± Δ/2, where Δ is the width of the 95% confidence interval (CI). One-way ANOVA followed by Tukey's post hoc test was used to calculate the significance of differences between the groups; *n* = 7 − 8.  ^∗^Significant difference vs. the control group without stress. ∧Significant difference vs. the control group with stress. Significance level:  ^∗,^∧*p* < 0.05;  ^∗∗∗,^∧∧∧*p* < 0.001.

The level of NO in the control brains taken from the stressed animals was significantly higher than in the naive control animals. In contrast, the levels of NO in stressed animals that were treated with IT were lower than in the brains of the stressed control group (they did not differ from the level determined in the naive control); in the case of the administration of the dose of 50 mg/kg b.w., statistical significance (*p* < 0.05) vs. the level of NO in the stressed control group was noted. Bupropion does not affect NO levels in the brain. In the brains of the bupropion-treated group, a comparable level of NO was detected as in the brains of the stressed control group. The results are shown in Figure 10(a).

There were no differences in TNF-alpha levels in brains collected from control and IT-treated animals. Significantly higher levels of TNF-alpha in comparison to both control groups were determined in the brain of mice treated with bupropion (*p* < 0.001). The results are shown in Figure 10(b).

## 4. Discussion

The present study shows for the first time that a single administration of hydroalcoholic leaf extract of *Isatis tinctoria* L. is able to reverse the behavioral response to restraint by a mechanism dependent, at least in part, on the modulation of oxidative stress, neuroinflammation, and NO reduction.

*Isatis tinctoria* is a plant with great therapeutic potential and extracts obtained from it possess the ability to inhibit the formation/activity of nitric oxide, anti-inflammatory, antioxidant, and neuroprotective properties [16, 39, 40]. Therefore, considering the above activities, preliminary pharmacological studies were performed to investigate the effect of polar extract from *I. tinctoria* leaves (IT) on stress-associated behaviors, as well as for the reduction of anxiety-like or depression-like behaviors. For this purpose, we used a validated model of acute restraint stress in mice [36, 41–43]. The latter is a widely used animal model to induce stress-related behaviors [36, 42], which are at least in part attributable to aberrations in the brain antioxidant and inflammatory systems [36, 44, 45].

Since most preclinical models target specific symptoms of psychiatric disorders [33], four behavioral tests were performed *in vivo*: the social interaction test, the dark/light box test, the splash test, and the locomotor activity test. These four behavioral tests are together suitable for phenotyping animal behavior that may be related to certain aspects of human behavior reflecting the behavioral aberrations in psychiatric conditions such as depression and anxiety [46].

Behavioral test results should always be interpreted considering the spontaneous activity of mice to ensure that the behavioral changes observed are not due to sedation or overstimulation after administration of the test extracts. Reducing spontaneous activity or overstimulation may contribute to false-positive or false-negative results. In this study, it was noticed that the intraperitoneal administration of IT at a dose of 50 mg/kg b.w. to nonstressed mice significantly reduces the spontaneous mobility of mice in comparison to the mobility determined in the control group, which indicates the sedative effect of the lowest dose of the extract tested. The plant extract is a mixture of various chemical compounds, and our research shows that in this lowest dose, we already have a sufficient presence of one ingredient to induce a significant sedative effect.

On the other hand, the lack of a sedative effect at higher doses may indicate that as the dose increases, the effects of other components of the extract cancel out this sedative effect. Further studies are planned to determine the specific compounds related to the effects detected in our research. However, a previous study [27] characterized the phenolic profile of polar extracts obtained from leaves of *I. tinctoria* by identifying Vicenin-2 and Isovitexin as the most abundant flavonoids. This analysis may suggest the hypothesis of the involvement of Vicenin-2 and Isovitexin in the activities reported by our experiment.

The downside is that the plant obtained from natural conditions may differ in composition depending on the substrate and the weather; therefore, after these preliminary tests with promising results, we plan to conduct research using the leaves of plants grown in the laboratory. If the biomass from in vitro cultures would match the level of plant activity, a material derived from in vitro cultures could be proposed as a homogeneous therapeutic material.

In the ARS model, stress caused a significant decrease in spontaneous mobility compared to the control group without stress. The mice, immobilized for 240 minutes, did not explore the activity cage to the same extent at the time of measurement as the unstressed mice. This may be due to, e.g., the immobilization-induced mental and physical fatigue and is a measurable symptom of induced stress. Administration of IT at a dose of 500 mg/kg b.w. in stressed mice significantly increased the activity of mice compared to stressed control mice. Thus, we can conclude that the highest tested dose of the extract, after which no significant effect on spontaneous mobility was observed in nonstressed mice, abolished spontaneous mobility disorders caused by acute stress. It should be noted that after intraperitoneal administration of IT at a dose of 50 mg/kg b.w. in stressed mice, no statistically significant reduction in mobility was observed compared to the control group without stress, despite the earlier determination of a significant sedative effect of the extract at this dose in mice without stress. The sedative effect observed at this dose (seen in unstressed mice), therefore, might have made a minor contribution to the improved stress response in this regard in stressed mice. The reference compound selected for the study, bupropion, significantly increased the spontaneous mobility of mice in naïve mice, which is consistent with the known stimulating and activating effect of this drug [47]. It was found that after applying both these doses of IT or bupropion, the spontaneous activity in the stressed mice did not differ from the spontaneous activity counted in the control mice without stress. Therefore, we conclude that as for bupropion, it could have been due to its stimulating effect, while the tested extract at a dose of 500 mg/kg or 50 mg/kg b.w. had a positive effect on these poststress disorders.

Social behavior disorders are frequently observed in people suffering from affective disorders such as depression and anxiety states [33]. The social interaction test was carried out to identify aberrations in social interactions in our animal model study. This test is used to study the interaction between two unknown mice in an open field [33]. The increase in the number of contacts may be due to the hyperactive behavior of the mouse. On the other hand, the reduced number of contacts may be related to the depressive or anxious behavior of the animals [48] and also result from sedation; therefore, the results are interpreted considering the effect on spontaneous activity. In a social interaction study performed, the number of times the test mouse approached the stimulating mouse was analyzed. In nonstressed mice, the extract administered at all doses and the reference compound did not influence social behavior. Even after administration of the extract at a dose of 50 mg/kg b.w., which caused sedation, no significant changes in the interaction between mice were observed in this test. The curiosity of the second subject was therefore stronger than that of the sedative effect, which is indeed a favorable result. It was observed, however, that the number of contacts initiated by stressed control mice is statistically significantly lower compared to contacts initiated by stress-free control mice, thus indicating a significant role that stress plays in impaired social functions. All the doses of IT tested, as well as the reference compound, bupropion, increased the number of approaches of the test mice to the stimulant mice (no statistical significance was also observed compared to stress-free control mice; therefore, the effect should be considered noticeable), and bupropion showed this effect is strongest; statistical significance was calculated compared to the stressed control group. This is probably due to the stimulating and activating effect of bupropion [47].

The dark/light box test was used to initially assess the anxiolytic effect of the test extracts. It is based on the rodent's innate aversion to brightly lit, open areas (mice like to spend time in small, sheltered spaces), and spontaneous exploratory behavior of new environments (mice placed in a new environment learn about their surroundings out of curiosity) [34]. In times of stress, a smaller, dark box is a safer place for mice compared to a larger, illuminated chamber, which they are reluctant to explore in this state. The dark/light box test is quick and easy to perform and requires no prior training of the animals. The anxiolytic effect of the extracts tested is indicated by an increase in spontaneous activity of mice, a decrease in the time spent in the dark part of the cage, and an increase in the number of passes through the opening between the light and dark chambers [34]. Therefore, the conducted light/dark box test focused on the analysis of three parameters: the number of passes through the opening between the light and dark field, activity in the bright field of the locomotive cage, and the time spent in the dark field during one entry into this part of the chamber. It was observed that the number of passes through the opening in the stressed control mice was statistically significantly lower than in the nonstressed control group. These results were in line with the results indicating sedation after a period of stress in the previously discussed test of spontaneous activity (stress reduced this activity). This reduction in the number of passages between boxes also proves the dullness of curiosity to explore by these animals and the reduction of the need to explore new spaces or the feeling of poststress anxiety.

It was also noted that in the ARS model, the extract was administered at a dose of 100 mg/kg b.w. did not significantly affect the number of passes between boxes; their number was comparable to the number of passes in stressed control mice. Therefore, no beneficial effect was shown at this dose. Administration of IT in doses of 500 mg/kg b.w. and 50 mg/kg b.w. caused more frequent passes of mice between light and dark fields compared to stressed control mice, with the number of passes being greater after administration of the extract at the highest dose. These results indicate the anxiolytic properties of the IT administered in the highest and the lowest concentration tested. As previously emphasized, the extract at a dose of 50 mg/kg b.w. in nonstressed mice induced a significant reduction in activity; therefore, it should be noted once again that in stressed mice, this sedative effect may be beneficial, which can be seen in the reduction of stress symptoms. The lack of an intermediate dose effect in this test, therefore, it can be explained that in this dose, the effect of the sedative compounds contained in the extract is already canceled out by the greater presence of compounds with the opposite activity in the extract. However, the composition of this dose does not yet cause anxiolytic effects noticeable after administering the highest dose of the extract.

Measurement of locomotor activity of mice in the light box after administration of all tested doses of extracts did not provide statistically significant results. However, the videos with the recordings of the experiment were watched, and it is clearly visible that the mice subjected to stress explored the cage less and sat in one place more often. However, they also made a significant number of movements while cleaning the fur (they visibly sweated during the stress induction period), and probably, these movements were also read by an automatic measuring device that recorded spontaneous activity in a bright box. Therefore, we conclude that the results were disturbed by the increased sweating during stress induction in the selected model and cannot be rightly interpreted.

However, when examining the time spent by mice in the dark box per entry, it was noticed that stressed mice spent statistically significantly more time in the dark box than control mice without stress. This is probably because naturally, dark places are perceived by mice as safer and nonthreatening, and mice under stress preferred to protect themselves there rather than explore the unknown, open, bright part of the box. All tested doses of IT, as well as the reference compound, bupropion, reduced the time spent by mice in the dark box during one stay compared to the time spent by the stressed control group. However, only the extract administered at a dose of 500 mg/kg b.w. shortened this time in a statistically significant way. This is further evidence of the anxiolytic effect of the highest tested dose of IT.

The splash test is used to assess anhedonia, which is one of the symptoms of depression [49]. Spraying the hair on the back of mice with a 10% viscous solution of sucrose induces grooming behavior, and the lack of it is considered an analogy of anhedonia [49]. In this study, the time to start washing, the total washing time, and the number of washes were measured, that is, parameters described in the literature as related to motivational behavior and self-care [49]. The test shows, however, that the administration of the extract in the maximum dose of 500 mg/kg b.w. to nonstressed mice, as well as bupropion, significantly extended the time to start washing. These are surprising observations, but in the case of bupropion, which increased spontaneous mobility at such a dose, it could have been due to agitation and the desire to visit a new space for mice, in which it was placed immediately after spraying, i.e., at the initial time of the test. While these two results appear logically complementary, the limitation of the study performed was the lack of a concurrent measurement of mobility, a test that could accurately indicate the dependence of not initiating cleaning on increased activity. However, more research is needed to explain the observation of this parameter regarding the effect of IT at the highest dose used.

Surprising results were obtained with mice in the ARS model. It turned out that subjecting the mice to stress did not cause a statistically significant extension of the time to start washing compared to nonstressed control mice, and the administration of IT at all doses tested did not significantly affect this time compared to stressed control mice. When analyzing the washing time, it was observed that stressed mice washed longer than nonstressed mice. The counts of the number of washes also provided results that were different from what was expected. Thus, inducing stress in mice resulted in more frequent grooming behavior compared to the stress-free control group. However, since all stressed mice sweat very significantly after the stress induction period, it could have a significant disruptive effect on the parameters determined in this test. Therefore, it should be concluded that the splash test is not suitable for evaluating depressive behaviors such as anhedonia after stress induction by temporary immobilization. Therefore, there is a real need to conduct another test in the future that will give unambiguous results.

Keeping in mind the knowledge that physical and mental stress activates cellular stress by intracellular pathways involved in increasing free radical production [50–52], we determined the effect of stress caused by immobilization as well as the effect of a single administration of IT on the level of ROS in the brain and the activity of antioxidant enzymes: SOD and CAT in brain and plasma. Our research showed that under the stress levels of antioxidant enzyme activity were significantly leveled after a single administration of IT. The most significant changes were observed after a dose of 500 mg/kg body weight and 50 mg/kg b.w., which correlates with the results of behavioral studies, because, as described above, it was the use of these doses that had a significant effect in reducing the symptoms of stress. It should be emphasized that the reduced CAT activity in the brain of stressed mice observed in the present study is in agreement with data previously reported by others [41, 43, 53]. Reduced CAT activity is an indicator of a prooxidative state since SOD converts superoxide anion to hydrogen peroxide (H_2_O_2_), but CAT does not metabolize H_2_O_2_ to water [54]. This excessive production of H_2_O_2_ may favor the Fenton reaction and the generation of hydroxyl radical, which in turn triggers lipid peroxidation. Therefore, we propose that one of the mechanisms of IT to reduce stress-induced behavior is to increase the ability to combat oxidative stress by influencing the activity of CAT (probably indirectly).

Nitric oxide is also known to modulate levels of cyclic guanosine monophosphate, which in turn induces a depression-like state in animals, reducing motivation to cope with stress [55]. In our study, we observed significantly increased levels of nitric oxide in the brain in stressed mice. However, what is worth emphasizing and may indicate a second mechanism of action in reducing poststress disorders, administration of IT reduced the level of NO in the brain, and it was best observed at the lowest dose used (50 mg/kg b.w.).

CRP is an acute phase protein that is widely used in clinical practice and has also been measured in many previous studies of behavioral disorders [56–58]. Additionally, it is known that elevated levels of TNF-alpha in the brain may be associated with local inflammatory pathways [56]. Recent data indicate that increasing plasma CRP concentrations in patients is also associated with decreased functional connectivity within reward circuitry and with high central nervous system glutamate level, which is correlated with symptoms of anhedonia [59]. In our study, we measured the level of CRP in the plasma taken from mice, as well as the level of TNF-alpha in the brains. In fact, it turned out that in stressed mice, plasma CRP levels increased significantly, but no significant changes in TNF-alpha levels were observed. These results may be because we performed an acute stress model, and the increased levels of TNF-alpha in the brain after this acute stress may not be visible enough yet. What is important, however, is the result obtained after administration of the IT, in doses reducing the symptoms of behavioral disorders, i.e., 500 and 50 mg/kg b.w., the level of CRP in the plasma was lower compared to the level determined in the stressed mice receiving only the vehicle (it did not differ from the level determined in unstressed mice). This underlines the importance of the anti-inflammatory effect of IT in reducing stress-induced disorders and may indicate a third mechanism of this action.

We used bupropion, an atypical antidepressant that inhibits the reuptake of noradrenaline and dopamine, as the reference compound in our research. It was effective in stress-initiated behavioral disorders (it had an activating and anxiolytic-like effect). Biochemical assays showed that bupropion also balanced the activity of antioxidant enzymes but did not affect the level of NO in the brain, and the marked markers of inflammation were even higher in the plasma and brain of the receiving group than in control animals. Bupropion increased the levels of BDNF in both the plasma and brain, which was not observed with IT. The stronger effect of reducing stress-induced behavioral disturbances after IT administration than after bupropion may therefore be the result of several mechanisms of action. Further extended studies are necessary to confirm and identify the specific target points of the action of the IT.

It is important to note that the *I. tinctoria* can be useful to treat stress-related disorders such as depression and anxiety and that this plant should be trialed in other conditions and disorders that are associated with immune-inflammatory and nitrooxidative stress processes and reduced neuroprotection. As such, extracts of this plant may be beneficial in preventing or treating suicidal behaviors [60], psychosis [61], mild cognitive impairment [62], and neurological disorders including Parkinson's and Alzheimer's diseases [63] and temporal lobe epilepsy [64].

## 5. Conclusion

These preliminary studies clearly show that *Isatis tinctoria* leaf extract may reduce stress-induced behavioral disturbances by regulating neurooxidative, neuronitrosative, and neuroimmune pathways. Therefore, extracts of this plant may be considered for further research on reducing stress-induced behavioral disorders. Confirmation of the beneficial properties of IT against stress-associated disorders is a first step and encourages research on plant material grown *in vitro* cultures, with a uniform and controlled metabolic profile, i.e., biochemically and/or genetically uniform biological material.

## Data Availability

The data presented in this study are available on request from the corresponding author.
